# Gentamicin Pharmacokinetics and Optimal Dosage in Infant Patients: A Case Report and Literature Review

**DOI:** 10.3390/ijerph192215360

**Published:** 2022-11-21

**Authors:** Hideo Kato, Mao Hagihara, Hiroko Matsuda, Takuya Iwamoto

**Affiliations:** 1 Department of Pharmacy, Mie University Hospital, Tsu 514-8507, Japan; 2 Department of Clinical Pharmaceutics, Division of Clinical Medical Science, Mie University Graduate School of Medicine, Tsu 514-8507, Japan; 3 Department of Molecular Epidemiology and Biomedical Sciences, Aichi Medical University Hospital, Nagakute 480-1195, Japan

**Keywords:** infant, gentamicin, pharmacokinetics

## Abstract

Gentamicin is an aminoglycoside antibiotic that is mostly used for the pediatric population. While the pediatric population is classified into neonates, infants, children, and adolescents based on developmental or maturational changes, infants are often overlooked in research. Three infant cases receiving gentamicin are presented to illustrate the pharmacokinetics and optimum dosage of gentamicin. Three infant patients received gentamicin (5.6–7.5 mg/kg/day) for urinary tract infections (UTIs) or bacteremia caused by *Enterobacter aerogenes*. The trough (Cmin) and peak (Cpeak) concentrations of gentamicin were 0.2–1.8 and 8.9 mg/L, respectively. The Cmin of a patient receiving gentamicin at 9.0 mg/kg/day was 3.3 mg/L, and the patient showed a decrease in urinary volume. The other two patients fully recovered from the infection and did not experience any adverse events. Additionally, we reviewed three studies regarding infant patients receiving gentamicin. The studies used gentamicin therapy for Gram-negative pathogen infections and UTIs caused by *Escherichia coli* and *Enterococcus faecalis*. The Cmin and Cpeak of patients receiving gentamicin at 2.2–7.5 mg/kg/day were 0.58–2.15 mg/kg and 4.67–8.88 mg/L, respectively. All patients were cured without any adverse events. Gentamicin dosages below 7.5 mg/kg/day may be effective and safe for use in infant patients. However, the optimal dosing regimen of gentamicin in infant patients is controversial, and limited data are available.

## 1. Introduction

Gentamicin is a bactericidal aminoglycoside antibiotic exerting activity against Gram-negative *Bacilli* and Gram-positive *Cocci* [1]. It remains a key antibiotic for the treatment of pneumonia and urinary tract infections (UTIs) in pediatric practice. However, most antibiotics, including gentamicin, were mainly developed for adults and subsequently extrapolated for dosing regimens in pediatric patients. Therefore, the optimal dosing regimen of gentamicin for pediatric patients remains controversial.

Pediatric populations exhibit developmental or maturational changes that may contribute greatly to the pharmacokinetic (PK) variability observed in pediatric patients [2,3]. Although the United States Food and Drug Administration (FDA) and the European Medicines Agency classify the pediatric population into neonates (birth to 1 month of life), infants (between 1 and 24 months), children (between 2 and 11 years), and adolescents (between 12 and 18 years) based on the complex changes and the anatomical, biochemical, and physiological differences related to age [4,5], infants are often overlooked as a subject of research. Previous PK studies have investigated neonates [6] and all age groups from neonates to children [7]. Here, we present three cases of infants treated with gentamicin.

## 2. Case Reports

Three infants who received gentamicin by intravenous infusion at Mie University Hospital between January 2015 and December 2021 were included in this study. The three infants fasted. The details of each infant case presented in this report are provided in Table 1. Gentamicin concentrations were measured by Dimension (Siemens Healthineers, Tokyo, Japan).

### 2.1. Case 1

**Figure 1:**
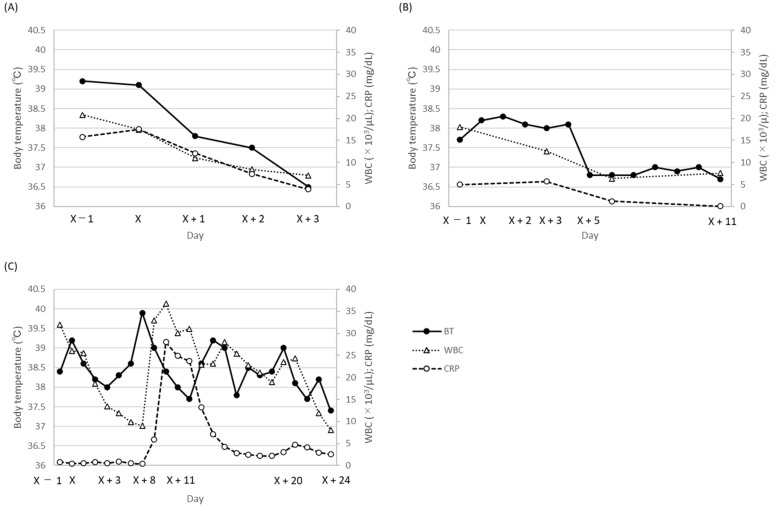
Time-dependent change in body temperature, WBC count, and C-reactive protein (CRP) levels during gentamicin therapy. (**A**) case 1; (**B**) case 2; (**C**) case 3. BT, body temperature; WBC, white blood cell; CRP, C-reactive protein.

A 5-month-old male infant was diagnosed with bilateral hydronephrosis, underwent nephrostomy one month before gentamicin therapy (day X – 28), and noted nephrostomy catheter obstruction a day before gentamicin therapy (day X − 1). On day X, purulent urine; high fever (39.1 °C); an elevated white blood cell (WBC) count (17,450 cells/μL; normal range, 3300–8600 cells/μL); and increased C-reactive protein (CRP) levels (17.53 mg/dL; normal range, 0–0.14 mg/dL) were observed. A diagnosis of UTI was made, and the patient (body weight: 5.3 kg) was administered intravenous gentamicin 7.5 mg/kg once daily immediately after one set of blood and urinary cultures were collected (day X). The next day (day X + 1), the trough concentration (Cmin) of gentamicin was 0.5 mg/L, but the gentamicin dosage remained the same as the starting dose. On day X + 2, the growth of *Enterobacter aerogenes* was observed in the urinary culture collected on day X (blood culture, negative), and the minimum inhibitory concentration (MIC) of the isolate against gentamicin and levofloxacin was less than 1 mg/L. The fever gradually subsided, and the WBC count and CRP levels gradually recovered to normal values (Figure 1A). On day X + 3, the nephrostomy catheter was removed because of defervescence (36.5 °C) and clear urine. Gentamicin was administered for four days, followed by oral levofloxacin for another three days. Renal function impairment (reduction in urinary volume: day X, 449 mL/day; day X + 3, 442 mL/day) was not observed during and after gentamicin therapy. No signs of UTI were observed after the completion of antibiotic therapy. During gentamicin therapy, PHYSIO 35 injection (electrolyte solution) was administered at an infusion rate of 2 mL/h.

### 2.2. Case 2

The second patient was a 7-month-old male infant (body weight: 6.4 kg) who showed signs of anuresis for the preceding two months. Since he presented with a high fever (38.2 °C) and turbid urine, a urinary culture was drawn (day X). Blood tests on day X − 1 showed an elevated WBC count (180,600 cells/μL) and CRP level (5.0 mg/dL). The patient was diagnosed with UTI, and empirical gentamicin at 2.3 mg/kg twice daily (day X) was administered. On day X + 2, *E. aerogenes* with a gentamicin MIC of less than 1 was detected in the urinary culture. The Cmin value of gentamicin was 0.8 mg/L on day X + 3, and the same gentamicin dosage was continued. Although the flow of clear urine was sufficiently established, a slight fever and a slight increase in the CRP level were observed. As the patient’s fever abated and laboratory data also improved (Figure 1B), on day X + 11 gentamicin therapy was discontinued. During gentamicin therapy, no reduction in the urinary volume was observed (day X, 541 mL/day; day X + 3, 578 mL/day; day X + 6, 602 mL/day; day X + 11, 590 mL/day). During gentamicin therapy, a SOLDEM 1 injection (glucose-electrolyte solution) was administered at an infusion rate of 10 mL/h.

### 2.3. Case 3

Case 3 was a 7-month-old female infant (body weight: 4.0 kg) with a history of pulmonary artery banding for tetralogy of Fallot, who had a high fever (38.4 °C) and presented blood in the stool (day X − 1). Following a clinical evaluation and cultivation test (blood culture, negative), the patient was diagnosed with a possible enteric infection, and gentamicin at 2.3 mg/kg three times daily was administered to target the Gram-negative pathogens in the intestine (day X). The Cmin values of gentamicin on day X + 3 and day X + 7 were 1.8 and 0.5 mg/L, respectively, and the gentamicin dosage was not changed. The WBC count and CRP level decreased till day X + 8, and blood feces were not noted from day X + 9. However, her body temperature started to increase from day X + 5, and her WBC count and CRP level also started to increase from day X + 9 (Figure 1C). On day X + 9, a set of blood cultures was collected, and methicillin-resistant *Staphylococcus aureus* (MRSA) with a gentamicin MIC of less than 1 (daptomycin MIC, unknown) was observed in the cultures on day X + 11. Moreover, the WBC count and CRP level raised sharply on day X + 11 (Figure 1C). As an antibiotic therapy, the gentamicin dosage was increased to 3.0 mg/kg three times daily, since the previous gentamicin dosage was thought not to have achieved the therapeutic Cpeak target (5–10 mg/L), and daptomycin 30 mg/day was added (day X + 11). The peak concentration (Cpeak) of gentamicin on day X + 12 was 8.9 mg/L. Since the Cmin value of gentamicin on day X + 13 was 3.3 mg/L, the gentamicin dosage was decreased to 3.0 mg/kg once daily on day X + 14. After gentamicin dosage reduction, the Cmin value of gentamicin decreased to 0.2 mg/L on day X + 24. Body temperature, WBC count, and CRP level gradually decreased, and the blood culture collected on day X + 20 was negative (day X + 25). Gentamicin and daptomycin were discontinued on day X + 24 and day X + 25, respectively. Regarding renal function, the urinary volume decreased from 655 mL/day on day X + 13 to 511 mL/day on day X + 15 but recovered to 581 mL/min on day X + 16. Except for the duration, the urinary volumes were constant (day X, 586 mL/day; day X + 3, 612 mL/day; day X + 7, 574 mL/day; day X + 24, 581 mL/day).

## 3. Literature Review

All literature published in PubMed, Cumulative Index to Nursing and Allied Health Literature, and Ichushi until June 2022 were identified by an electronic search, using the following terms: “gentamicin”, “infant”, “trough”, and “peak”. Exclusion criteria were as follows: (i) reviews, (ii) papers describing a study already included, (iii) non-clinical studies, and (iv) papers containing no data regarding gentamicin concentrations. The database searches yielded three out of 300 candidate studies [8,9,10], two PK studies [8,10], and one case-control study [9] (Table 2). All were single-center studies. Data from 24 infants have been reported. Two articles reported gentamicin therapy for Gram-negative pathogenic infections [8] and UTIs due to *Escherichia coli* and *Enterococcus faecalis* [9]. None of the patients were treated with other antibiotics. The range of gentamicin doses was 2.0–2.5 mg/kg, and gentamicin was administered one to three times per day (2.2–7.5 mg/kg/day). The range of Cpeak values was 4.67–8.88 mg/L, and the range of Cmin values was 0.58–2.15 mg/L. In contrast, no studies have reported the MIC of the isolated bacteria. One article reported clinical and bacteriological effects [9], and all patients fully recovered from the infection. None of the patients experienced any adverse events in these two studies [9,10].

## 4. Discussion

The literature review showed that the PK of gentamicin in infants has not been well investigated. In particular, the lack of knowledge on Cpeak/MIC was highlighted. Moreover, we suggest a potential candidate for optimal dosage in infant patients based on the association between the gentamicin dosage and MICs presented in our cases and previous studies.

Gentamicin is used as a combination therapy with beta-lactam antibiotics. A previous study in Japan reported that gentamicin monotherapy is administered in 33.2% of patients with UTIs and 11.8% of patients with bacteremia [11]. In the present study, three case were treated with gentamicin monotherapy due to its bacteriological effect against Gram-negative *Bacilli* and Gram-positive *Cocci*.

Gentamicin in the pediatric population is administered either in a conventional dosing regimen of twice or thrice daily or in an extended dosing regimen of once daily in a clinical setting [12]; thus, consistent dosing schedules have not been reported in previous studies or in the present cases [8,9,10]. Moreover, the cases receiving a gentamicin dosage below 7.5 mg/kg/day in the previous and present studies were cured with no adverse events. Therefore, a gentamicin dosage below 7.5 mg/kg/day may be effective and safe for use in infant patients. However, the optimal dosing regimen of gentamicin in infant patients is controversial.

PK in pediatric patients is affected by age. Changes in body composition and organ maturation during the first two years of life should be considered. The glomerular filtration rate (GFR) depends on the functional capacity of the kidneys and renal blood flow, which increases from 12 mL/min at birth to 140 mL/min in the first two years of life [13]. The distribution of gentamicin is mainly influenced by body fluid composition, which rapidly changes during the first two years of life [13]. Consequently, a higher clearance and smaller volume of distribution of gentamicin are expected in infants compared to neonates. GFR then decreases to approximate adult values as the child grows into adolescence [14]. Therefore, the differences in PK between infants and other parts of the pediatric population justify the need to perform extensive PK studies in infants.

Gentamicin is mainly eliminated through renal excretion. As the renal function (urinary volume) decreased, the Cmin increased in Case 3. In the present study, the key factor in gentamicin therapy was found to be renal function. In particular, urinary volume may be an appropriate index for assessing renal function.

The Cpeak of gentamicin was used to determine its efficacy, and Cmin was used to define its toxicity [15]. The therapeutic Cpeak and Cmin targets are 5–10 mg/L and less than 1 mg/L, respectively [16,17]. Among the cases and studies that reported Cpeak values, all patients achieved the Cpeak target and fully recovered from infection. In contrast, although 6 of the 12 patients showed a Cmin of over 1 mg/L, no adverse events were observed. The Cmin of patients who received gentamicin at 9.0 mg/kg/day was 3.3 mg/L, and mild renal function impairment was observed. The CL and Ke under a gentamicin dosage of 9.0 mg/kg/day were lower than those of patients in the previous studies [8,9,10]. Therefore, a dose of 9.0 mg/kg/day may deteriorate renal function. In contrast, the Cmin of the patient receiving a high dose of 7.5 mg/kg once daily in our study was less than 1 mg/L. Some meta-analyses have been reported to establish evidence for PK/PD. One meta-analysis reported the Cmin of gentamicin for reducing the risk of toxicity in neonates [18]. The results demonstrated that a Cmin of less than 2 mg/L decreased the risk of toxicity caused by gentamicin; thus, a gentamicin dose of 7.0 mg/kg once daily can be used to achieve a Cmin of less than 2 mg/L in the pediatric population [19]. Recently, the optimal gentamicin dosage in different pediatric age groups was investigated using population PK and Monte Carlo simulations [20]. Infants require daily doses of at least 4.0–8.0 mg/kg. Therefore, the gentamicin tolerability in infants may be similar to that in neonates. Single or multiple administrations of the recommended dose may be sufficient to attain a Cpeak of over 5 mg/L and a Cmin of less than 2 mg/L. However, the Cmin may exceed 2 mg/L, and the frequency of adverse events may increase in cases where the daily dose of gentamicin exceeds 8.0 mg/kg.

## 5. Conclusions

In conclusion, despite the very limited number of cases investigated and articles reviewed, this study provides a basic overview of gentamicin therapy for infant patients and the challenges to be addressed in the future, especially the optimum Cpeak/MIC ratio of gentamicin for infant patients. In terms of Cpeak and Cmin, further large-scale studies are necessary to obtain more robust evidence. The data provided in this study may aid in optimizing gentamicin treatment for infant patients.

## Figures and Tables

**Table 1:** Characteristics of infants treated with gentamicin.

Case No.	Sex	Age (Month)	Body Weight (kg)	Urinary Volume (mL/Day)		Scr (mg/dL)	Type of Infection	Bacteria		MIC of GM (mg/L)
1	Male	5	5.3	266		0.21	UTI	*Enterobacter aerogenes*		≤1
2	Male	7	6.4	541		0.22	UTI	*Enterobacter aerogenes*		≤1
3	Female	7	4.0	386		0.39	Bacteremia	MRSA		≤1
**Case** **No.**	**Regimen**	**Treatment** **Duration** **(Day)**	**Sampling Time after GM Administration**	**Cpeak** **(mg/L)**	**Cmin (mg/L)** **Mean ± SD**	**CL** **(mL/min/kg)**	**Ke** **(h^−1^)**	**Clinical** **Response**	**Bacteriological** **Effect**	**Adverse** **Event**
1	7.5 mg/kg × 1/day (7.5 mg/kg/day)	4	Cpeak, NA; Cmin, 24 h	NA	0.5	NA	NA	Improvement *	Eradication	None
2	2.3 mg/kg × 2/day (4.6 mg/kg/day)	12	Cpeak, NA; Cmin, 12 h	NA	0.8	NA	NA	Cure	Eradication	None
3	1. 2.3 mg/kg × 3/day (6.9 mg/kg/day) 2. 3.0 mg/kg × 3/day (9.0 mg/kg/day) 3. 3.0 mg/kg × 1/day (3.0 mg/kg/day)	25	Cpeak, 1 h; Cmin, 8 h	1. NA 2. 8.9 3. NA	1. 1.8; 0.5 2. 3.3 3. 0.2	0.91	0.12	NA	Eradication	None

CL, clearance; GM, gentamicin; h, hour; Ke, elimination rate; MIC, minimum inhibitory concentration; MRSA, methicillin-resistant *Staphylococcus aureus*; NA, not available; Scr, serum creatinine clearance; SD, standard deviation; UTI, urinary tract infection. Clinical response to gentamicin therapy was classified as cure, improvement, or failure, each of which was judged by physicians. * An additive effect might have influenced the clinical effect at the completion of antibiotic therapy.

**Table 2:** Characteristics of infants treated with gentamicin in previous studies.

Study	Study Design	Setting	No. of Patients	Age (Month)	Body Weight (kg)	Renal Function	Type of Infection		Bacteria	MIC of GM (mg/L)
Sunagawa K, 1983	PK study	Single-center	6	Median 8.6 (2.0–21.0)	Median 5.2 (4.1–9.7)	NR	Gram-negative pathogen infection		Gram-negative pathogens	NR
Sakata H, 1988	Case-control study	Single-center	3	Median 4.0 (2.0–4.0)	Median 6.9 (5.4–7.6)	NR	UTI		*Escherichia coli* and *Enterococcus faecalis*	NR
Moffett BS, 2010	PK study	Single-center	15	Median 3.7 (1.2–10.8)	NR	CCr, 81.1 ± 29.2 mL/min 1.73 m^2^	NR		NR	NR
**Study**	**Regimen**	**Treatment** **Duration** **(Day)**	**Sampling Time after GM Administration**	**Cpeak** **(mg/L)** **Mean ± SD**	**Cmin** **(mg/L)** **Mean ± SD**	**CL** **(mL/min/kg)** **Mean ± SD**	**Ke** **(h^−1^)** **Mean ± SD**	**Clinical** **Effect**	**Bacteriological** **Effect**	**Adverse** **Event**
Sunagawa K, 1983	Mean ± SD 2.5 ± 0.2 mg/kg × 1/day; (2.2–2.7)	NR	Cpeak, 1 h; Cmin, 6 h	6.26 ± 1.11 (5.09–7.82)	1.30 ± 0.48 (0.83–2.15)	2.91	0.39	NR	NR	NR
Sakata H, 1988	2.0 mg/kg × 3/day 2.5 mg/kg × 2/day 2.0 mg/kg × 2/day	Median 7 (5–7)	Cpeak, 0.5 h; Cmin, 12 h	6.87 ± 1.64 (4.67–8.88)	0.80 ± 0.34 (0.58–1.20)	2.39 ± 0.35 (2.07–2.76)	0.35 ± 0.06 (0.28–0.40)	All patients cured.	Eradication, *n* = 2; Unknown, *n* = 1	None
Moffett BS, 2010	2.5 mg/kg × 3/day	NR	Cpeak, 1 h; Cmin, 8 h	7.40 ± 1.70	1.30 ± 0.50	1.50	0.28	NR	NR	None

CCr, creatinine clearance; CL, clearance; GM, gentamicin; h, hour; Ke, elimination rate; MIC, minimum inhibitory concentration; NR, not reported; PK, pharmacokinetic; SD, standard deviation; UTI, urinary tract infection.

## Data Availability

All data are included in this article.
